# Comparing febrile children presenting on and off antibiotics to the emergency department: a retrospective cohort study

**DOI:** 10.1186/s12887-020-2007-4

**Published:** 2020-03-12

**Authors:** R. D. Sawaya, T. El Zahran, S. Mrad, C. Abdul Massih, S. Shaya, M. Makki, H. Tamim, M. Majdalani

**Affiliations:** 1 Department of Emergency Medicine, American University of Beirut Medical Center, Beirut, Lebanon; 2 Department of Emergency Medicine, Henry Ford University, Detroit, MI USA; 3 Clinical Research Institute, American University of Beirut Medical Center, P.O. Box 11-0236, Riad El-Solh/ Beirut, 1107 2020 Lebanon; 4 Department of Pediatrics, American University of Beirut Medical Center, Beirut, Lebanon

**Keywords:** Febrile children, Serious bacterial infections, Antibiotic use

## Abstract

**Background:**

It is not yet known how antibiotics may affect Serious Bacterial Infections (SBI). Our aim is to describe the presentation, management, and serious bacterial infections (SBI) of febrile children on or off antibiotics.

**Methods:**

Retrospective, cohort study of febrile Emergency Department patients, 0–36 months of age, at a single institution, between 2009and 2012.

**Results:**

Seven hundred fifty-three patients were included: 584 in the No-Antibiotics group and 169 (22%) in the Antibiotics group. Age and abnormal lung sounds were predictors for being on antibiotics (OR 2.00 [95% CI 1.23–3.25] and OR 1.04 [95% CI 1.02–1.06] respectively) while female gender, and lower temperatures were negative predictors (OR 0.68 [95%0.47–0.98] and OR 0.47 [95% CI 0.32–0.67] respectively). Antibiotics were prescribed by a physician 89% of the time; the most common one being Amoxicillin/Clavulanic Acid (39%). The antibiotic group got more blood tests (57% vs 45%) and Chest X-Rays (37% vs 25%). Overall, the percent of SBIs (and pneumonias) was statistically the same in both groups (6.5% in the No-antibiotic group VS 3.6%).

**Conclusions:**

Children presenting on antibiotics and off antibiotics were significantly different in their presentation and management, although the overall percentages of SBI were similar in each group. Further investigations into this subgroup of febrile children are needed.

## Background

Children with fever constitute a substantial proportion of ambulatory emergency department (ED) visits [1]. Serious bacterial infection (SBI) rates are still elevated: up to 12.8% in febrile infants less than 60 days of age [2], and up to 7.2% in children less than 5 years of age [3]. In the 1990s, several studies developed prediction rules to identify SBI in febrile infants [4–7]. Many have been revisited as the bacterial landscape has changed especially with the advent of vaccines [8–11]. However, as antibiotic use may alter the patients’ microbiome [12] and test results [13], including cultures [14], febrile children on antibiotics are typically excluded from studies on SBI [4–11]. In fact, there is no data describing febrile children presenting to the ED on antibiotics, nor the type of SBIs they may present with. Therefore, it is unclear how to use the data on SBI predictors and diagnosis in this subpopulation of febrile children already.

The objective of this study was to describe previously healthy children, presenting to the ED with fever, stratified by previous antibiotic use or not; and to describe the distribution and types of SBI in those two groups.

## Methods

### Study design

We carried out a retrospective, cohort study of patients 0–36 months of age presenting with fever to the ED of the American University of Beirut Medical Center in Beirut, Lebanon, between July 1, 2009 and June 30, 2012. Institutional Review Board approval was obtained. This is an ED of a tertiary care center, in a middle-income country where pediatric patients during the time of our data collection where seen primarily by pediatricians with or without intensive care training.

### Population

We included all patients 0 to 36 months of age, with fever (rectal temperature ≥ 38 °C or ≥ 37.6 °C by any other route) measured in the ED, at home or at the pediatrician’s office. We retrieved the records of patients with one or more of the following chief complaints, ED discharge diagnoses or hospital admission/discharge diagnoses: fever, cough, sore throat, runny nose, fussy, lethargy, decreased activity, seizure activity, vomiting, diarrhea, pneumonia, urinary tract infection (UTI), viral illness, tonsillitis, pharyngitis, cellulitis, abscess, meningitis, encephalitis, sepsis, septic shock, bacteremia.

We excluded all patients with an underlying immunosuppressive disease or immunosuppressive medication; with an underlying chronic disease (that may impact fever management); with a previous UTI; and admitted to the ED or hospital within the last 2 weeks.

### Data collection

We included information on: patient demographics, clinical presentation, and management. Data was collected by 4 physicians who had a training by the principle investigator in order to use the same terminology and categorize signs and symptoms in the same way.

## Definitions

We defined Serious Bacterial Infection (SBI) as one of the following:

1. Urinary Tract Infection: a positive urine culture > 5000 cfu/ml for suprapubic aspiration (SPA), > 10,000 cfu/ml for a sterile catheterization in children < 2 months old; > 50,000 cfu/ml AND pyuria by urinalysis (WBC > 5/mm^3^) by sterile catheterization or SPA and > 100,000 cfu/ml for clean catches [2, 15].
2. Bacteremia: a positive bacterial culture with a true pathogen other than Coagulase Negative Staphylococcus or other commensal bacteria (such as Staphylococcus epididymis and Diphteroid), which were considered contaminants unless treated as true infections per documentation [2, 7, 16, 17].
3. Meningitis: a positive cerebrospinal fluid culture other than coagulase negative Staphylococcus which was considered a contaminant, unless treated as true infections per documentation [2, 18].

We defined Pneumonia as a Chest X-Ray reported by a radiologist as definite or probable for a pneumonia (“Infiltrate”, “consolidation” or “concerning for developing pneumonia”) irrespective of microbiological results as these are low yield [19]. This definition reflects clinical practice.

We defined tachypnea and tachycardia as values above the upper limit of normal for age, as per Additional file 1. We defined hypoxia as an oxygen saturation ≤ 97%.

We defined abnormal perfusion as any documentation of mottled skin, or capillary refill greater than 3 s, or a flash capillary refill consistent with possible warm shock.

The “Antibiotic” group included all children who were on antibiotics prior to the ED visit as per care giver’s report or who had received antibiotics within the past 2 weeks. The “No-Antibiotic” group included the children who had not received any antibiotics prior to the ED visit.

### Statistical analysis

The Statistical Package for Social Sciences (SPSS), version 24.0 was used for data cleaning, management and analyses. Descriptive statistics were summarized by presenting the number and percentage for categorical variables, whereas continuous ones were presented by mean and standard deviation (±SD). In the bivariate analysis, the association between antibiotic use and other categorical variables was assessed using Chi-Square test, whereas Student’s t-test was used for the association with continuous variables. Multivariate regression analysis was used to adjust for potentially confounding variables. Variables which were statistically significant in the analysis or clinically important were included in the multivariate analysis. The stepwise logistic regression analysis assessed the association between antibiotic use and the different predictors. *P*-value of 0.05 was set for the entry of potential predictors into the model, whereas a *p*-value of 0.1 was set for removal from the model. The results were presented by the odds ratio (OR) and 95% confidence interval (CI). *P*-value of < 0.05 was considered statistically significant. Missing data was left empty.

The datasets analyzed during the current study are available from the corresponding author on reasonable request.

## Results

**Fig. 1:**
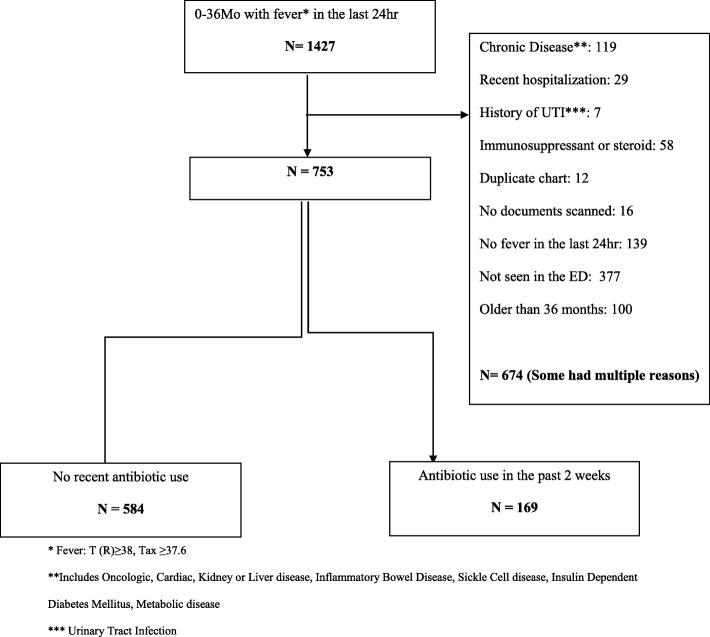
Flow chart of included patients. * Fever: T (R) ≥ 38, Tax ≥37.6. **Includes Oncologic, Cardiac, Kidney or Liver disease, Inflammatory Bowel Disease, Sickle Cell disease, Insulin Dependent Diabetes Mellitus, Metabolic disease

We retrieved 1427 patients from the medical records; 753 met our inclusion criteria and were analyzed: 584 in the No-Antibiotics group and 169 (22.4%) in the Antibiotics group (Fig. 1).

As per Table 1, children in the Antibiotic group were significantly older (21.2 months ±9.2 compared to 16.9 ± 10.3; *p* < 0.0001), in fact none of the children < 90 days of age had received antibiotics prior to presentation in this sample. In addition, the Antibiotic group was mostly of male gender (62.7% compared to 51.4%, *p* < 0.009), and had a longer duration of fever prior to presentation (4.5 days ±5.5 compared to 2.1 ± 1.6, *p* < 0.0001). Interestingly, associated symptoms presented as frequently in both groups except for a sore throat: 10.1% in the Antibiotic group compared to 4.8% in the No-Antibiotic group (*p* = 0.01) (Table 1).

**Table 1:** Variables in the **history and physical exam** associated with patients 0–36 months of age presenting to the ED with fever, previously on and off antibiotics

	All *N* = 753	AB- *N* = 584	AB+ *N* = 169	*P*-Value
**Age (months),** mean(±SD)	17.8 ± 10.2	16.86 ± 10.26	21.22 ± 9.18	< 0.0001
**Male**	406 (53.9)	300 (51.4)	106 (62.7)	0.009
**Fever Duration (days),** mean(±SD)	2.9 ± 3.5	2.14 ± 1.64	4.55 ± 5.54	< 0.0001
**Congestion,** yes	251 (33.4)	196 (33.6)	55 (32.7)	0.83
**Immunizations up to date for age,** yes	259 (97.7)	235 (97.5)	24 (100.0)	1.00
**At least one of the below symptoms**	664 (88.4)	509 (87.3)	155 (92.3)	0.07
Respiratory Symptoms	416 (55.4)	323 (55.4)	93 (55.4)	0.99
Gastrointestinal Symptoms	332 (44.2)	253 (43.4)	79 (47.0)	0.40
Urinary Symptoms	11 (1.5)	10 (1.7)	1 (0.6)	0.47
Sore Throat	45 (6.0)	28 (4.8)	17 (10.1)	0.01
Otalgia	31 (4.1)	25 (4.3)	6 (3.6)	0.67
Rash	36 (4.8)	29 (5.0)	7 (4.2)	0.67
Decreased Appetite	285 (38.2)	223 (38.6)	62 (36.9)	0.69
Decreased Urine output	67 (9.3)	56 (10.1)	11 (6.5)	0.16
Decreased Activity	120 (16.3)	94 (16.5)	26 (15.5)	0.75
Change in mental status	118 (15.7)	84 (14.4)	34 (20.2)	0.07
**ED Tmax**^a^**(°C)** < 38	243 (32.3)	161 (28.4)	83 (49.7)	< 0.0001
38–39.4	353 (46.9)	298 (52.6)	55 (33.3)	
> 39.5–40	99 (13.1)	78 (13.8)	21 (12.7)	
> 40	37 (4.9)	30 (5.3)	7 (4.2)	
**Tachycardia** (*n* = 291)	201 (69.1)	75 (53.2)	126 (84)	< 0.0001
**Tachypnea** (*n* = 275)	26 (9.5)	10 (7.4)	16 (11.5)	0.24
**Hypoxia** (*n* = 444)	75 (20.4)	55 (15.8)	20 (20.6)	0.24
**Normal physical exam**	732 (97.6)	568 (97.3)	164 (98.8)	0.39
At least one abnormal finding below	21 (2.4)	16 (2.7)	5 (1.2)	0.39
Sick Looking	28 (37)	28 (4.8)	0 (0.0)	0.004
Abnormal lung sounds	92 (12.6)	59 (10.4)	33 (20.1)	0.001
Lungs wheezing	36 (4.9)	29 (5.1)	7 (4.3)	0.66
Abnormal mental status ^b^	81 (10.8)	71 (12.2)	10 (6.0)	0.02
Abnormal perfusion (*n* = 19)	12 (63.2)	12 (70.6)	0 (0.0)	0.12
Abnormal TM ^c^	155 (20.7)	110 (18.8)	45 (27.1)	0.02
Abnormal tonsils	380 (50.8)	283 (48.5)	97 (59.1)	0.02
Skin rash	38 (5.2)	34 (5.9)	4 (2.4)	0.07
**Well appearing baby**^d^	85 (11.3)	72 (12.3)	13 (7.7)	0.09

Percentages are of the total who had a response to that finding

^a^Highest temperature measured in the Emergency Department

^b^Includes hypoactivity, lethargy, sleepy, irritable

^c^*TM* Tympanic membrane

^d^normal physician exam and no symptoms other than congestion

The specifics of the antibiotic use within 2 weeks prior to presentation to the ED with a fever were quite varied in the Antibiotic group. The majority, (82%) were still takings antibiotics at presentation; and 10.8% were taking multiple. The mean days of antibiotic use was 3.5 ± 3.0 days. The antibiotic was prescribed by a Medical Doctor in 89.3% (101/113) of the cases. Finally, the most common antibiotic used was an oral 3rd generation cephalosporin at 33.2% followed by a combination of penicillin/beta-lactamase inhibitor at 31.9%.. Interestingly, up to 10.2% had received intravenous (IV) or intramuscular (IM) 3rd generation cephalosporin,, prior to the ED visit.

When comparing the two sub-groups (Table 1), we noted that the Antibiotic group was more likely to be tachycardic (84% compared to 53.2%; *p* < 0.0001); to have abnormal lung sounds (20.1% compared to 10.4%; *p* = 0.001), an abnormal tympanic membrane (27.1% compared to 18.8%; *p* = 0.02); and abnormal tonsils (59.1% compared to 48.5%; *p* = 0.02). While the No-Antibiotic group were more likely to have an abnormal mental status (12.2% compared to 6%; *p* = 0.02) and to be looking more sickly (4.8% compared to 0; *p* < 0.001).

The Antibiotic group was more frequently tested by blood work (56.8% compared to 45.0%, *p* = 0.01) and chest radiography (37.3% compared to 24.7%, *p* = 0.001) (see Table 2). But when tested, the No-Antibiotic group had more bandemia than the Antibiotic group (mean 0.9 ± 5.0 compared to 0.1 ± 0.6, *p* = 0.02); a more frequently positive urine analysis (positive leukocyte esterase in 31.8% compared to 9.4%, *p* = 0.01 and positive for WBCs in 23.8% compared to 6.3%, *p* = 0.03) and to have influenza (*p* = 0.03). Interestingly, the frequency of fluid boluses and admissions was the same in both groups.

**Table 2:** Variables in the **management/results** associated with all patients 0–36 months of age presenting to the ED with fever, previously on and off antibiotics

Management & results	All *N* = 753	AB- *N* = 584	AB+ *N* = 169	*P*-Value
**Any blood work,** yes	359 (47.7)	263 (45.0)	96 (56.8)	0.01
Blood WBC, mean(±SD)	13,468 ± 6363	13,324 ± 6318	13,865 ± 6502	0.48
Blood Neutrophils, mean(±SD)	53.1 ± 17.0	52.50 ± 16.93	54.58 ± 17.07	0.30
Blood Bands, mean(±SD)	0.7 ± 4.3	0.86 ± 4.97	0.09 ± 0.65	0.02
Blood lymphocytes, mean(±SD)	36.0 ± 16.1	36.48 ± 16.08	34.81 ± 16.24	0.39
Blood CRP, mean(±SD)	52.3 ± 68.7	52.35 ± 70.21	52.03 ± 65.11	0.97
**Bacteremia** (*n* = 156 for blood cultures)	4 (2.6)	2 (1.8)	2 (4.7)	0.31
**Any urine test done,** yes	168 (22.3)	133 (22.8)	35 (20.7)	0.57
Urine LE -positive	44 (27.3)	41 (31.8)	3 (9.4)	0.01
Urine Nitrites -positive	16 (9.9)	15 (11.6)	1 (3.1)	0.20
Urine bacteria -positive	29 (19.0)	25 (20.7)	4 (12.5)	0.30
Urine WBC -positive	33 (20.4)	31 (23.8)	2 (6.3)	0.03
**UTI** (*n* = 156 for urine cultures)	40 (25.6)	36 (28.8)	4 (12.9)	0.07
**CXR done,** yes	207 (27.5)	144 (24.7)	63 (37.3)	0.001
**Positive/probable for pneumonia**	110 (53.1)	73 (50.7)	37 (58.7)	0.25
**Any CSF tested,** yes	37 (4.9)	37 (6.3)	0 (0.0)	0.001
CSF WBC -positive	7 (25.9)	7 (25.9)	0 (0.0)	NA
**CSF culture** -positive	2 (5.9)	2 (5.9)	0 (0.0)	NA
**Throat culture done,** yes	59 (7.8)	39 (6.7)	20 (11.8)	0.03
Throat culture - positive	4 (3.0)	4 (3.5)	0 (0.0)	1.00
**Stool studies,** yes	68 (9.0)	46 (7.9)	22 (13.0)	0.04
Stool culture -positive	1 (5.3)	1 (5.3)	0 (0.0)	NA
**Nasopharynx,** yes	94 (16.1)	94 (16.1)	0 (0.0)	NA
RSV -positive	15 (34.1)	12 (36.4)	3 (27.3)	0.72
Flu -positive	13 (21.3)	13 (28.3)	0 (0.0)	0.03
**None of the above testing done**^a^	266 (35.3)	212 (36.3)	54 (32.0)	0.30
At least 1	487 (64.7)	372 (63.7)	115 (68.0)	
**None tested for SBI**^b^	543 (72.1)	427 (73.1)	116 (68.6)	0.25
At least 1 for SBI	210 (27.9)	157 (26.9)	53 (31.4)	
**Fluid bolus 20 ml/Kg,** yes	82 (11.1)	60 (10.5)	22 (13.0)	0.35
**Admission**	154 (20.5)	121 (20.7)	34 (19.5)	0.74

^a^testing done to look for signs of SBI or other infectious causes; *WBC* White blood cells, *LE* Leukocyte Esterase, *UTI* Urinary tract infection, *CXR* Chest Radiography, *CSF* cerebrospinal fluid

^b^Blood culture done, or Urine culture done, or CSF culture done

In the multivariate analysis reported in Table 3, age, and abnormal lung sounds were predictors for being on antibiotics. In fact, each 1 month increase in age increased the odds of being on antibiotics by 1.04 (95% CI: 1.02–1.06). Finally, of all the patients, 5.8% had at least one SBI. When analyzed by Antibiotic vs. No-Antibiotic group, the number of SBIs remained similar with no statistical difference (*p* = 0.15). However, UTIs were statistically more common in the No-Antibiotic group (12.5 and 21.9%; *p* = 0.002 and 6.2 and 2.4%; *p* = 0.05, respectively) (Table 4). Our data on bacteremia and meningitis were too few to analyze further. Since there were no children < 90 days old on antibiotics, we did not do any subgroup analysis for this age in this comparative study.

**Table 3:** Multivariate analyses to identify the predictors of presenting to the ED after **being on antibiotic** in the past 2 weeks

Antibiotic		
	OR (95% CI)	*P*-Value
**Age (months)**	1.04 (1.02–1.06)	< 0.001
**Gender -**Female	0.68 (0.47–0.98)	0.037
**Height of fever in the ED,** 38–39.4	0.47 (0.32–0.67)	< 0.001
**Abnormal lung sounds**	2.00 (1.23–3.25)	0.005

Variables included in the model were:

Age, Gender, Sore throat, Symptoms, Height of fever in the ED (reference: < 38), Abnormal lung sounds, Lungs wheezing, Abnormal mental status, Abnormal Tympanic Membranes, Abnormal tonsils, Skin rash, Normal physical exam, Well appearing baby (reference: no)

**Table 4:** Number of Serious Bacterial Infection (SBI) **in all** patients 0–36 months of age, tested or not for SBI

SBI variable	All *N* = 753	AB- *N* = 584	AB+ *N* = 169	*P*-Value
**Urinary Tract Infection** -positive	40 (5.3)	36 (6.2)	4 (2.4)	0.05
**Bacteremia** -positive	4 (0.5)	2 (0.3)	2 (1.2)	0.22
**Meningitis** -positive	2 (0.3)	2 (0.3)	0 (0.0)	1.00
**Negative for any SBI**	709 (94.2)	546 (93.5)	163 (96.4)	0.15
**At least 1 SBI positive**	44 (5.8)	38 (6.5)	6 (3.6)	

## Discussion

Children presenting with antibiotics to the ED are usually excluded from studies on febrile children. Our study is the first to describe febrile children on antibiotics. In our sample, a third of the febrile, healthy children presenting to the ED were already on antibiotics. These were significantly different than the group off antibiotics and were managed slightly differently. Interestingly, the overall percentages of SBIs were similar in each group, so were the admission and IV fluid bolus rates.

We found that older age, female gender, fever and abnormal lung sounds in the ED, were predictors of being on antibiotics prior to the visit. In addition, we showed that the antibiotic group had more focal infections (lungs, tonsils, and ears) and was perhaps started on antibiotics for that reason; this may be explained by the fact that upper respiratory infections (URTI) are the most common reason for being on outpatient antibiotics [20]. The No-Antibiotic group however did not have apparent focal infections but when they presented to the ED, they were sicker. Yet our overall rate of at least one SBI (Bacteremia, Meningitis and UTI) was 5.8% without reaching any significant difference when comparing both sub-groups on and off-antibiotics. This may be an underestimation in the Antibiotic group if these affected cultures; however, this also reflects the daily practice we face in the ED. Given the similar rates of SBI in both groups, and one study noting that antibiotics may in fact prevent complications in certain infections such as URTI, pharyngitis, otitis and hence have a protective effect [21], further studies looking at the clinical impact of this antibiotic use are needed.

The most common source of antibiotic prescription in our country remains the physician but only at 89.3%. It is worth noting that our Lebanese pharmacies can still issue an antibiotic without a prescription. This fits with results from a recent Lebanese and Middle Eastern report that antibiotics were one of the most common medications self-prescribed by patients [22, 23].

Finally, in our sample, the most common antibiotics used were broad spectrum antibiotics, such as a 3rd generation cephalosporin. As we noted that most patients on antibiotics had abnormal lung sounds, tympanic membranes or tonsils, perhaps these were to treat a pneumonia, otitis or Streptococcus tonsillitis. This is an interesting choice given the American Academy of Pediatrics (AAP) guidelines to treat these primarily with Amoxicillin [24–26]. However, the use of a combined penicillin/beta-lactam inhibitor does follow local patterns of streptococcus pneumonia resistance to Amoxicillin [27, 28] but is not justified for Streptococcus tonsillitis. In addition, it is important to note that 10% had a parenteral form prescribed. Lack of adherence to antibiotic use guidelines has already been documented in Lebanon [28, 29] The above information on antibiotic use and misuse begs for national campaigns for antibiotic stewardship including guidelines and education A 2016 study of Lebanese hospitals showed that only 7% knew what the term antimicrobial stewardship meant, although around 65% reported having some type of antibiotic control program in the hospital and only 50% had an outcome measure in place [30]. However, in recent years, The Lebanese Society of Infectious Diseases has published several articles guiding the treatment of specific diseases such as UTIs and complicated intraabdominal infections [31, 32]. .Moreover, the Alliance for the Prudent Use of Antibiotics (APUA) has a Lebanese chapter that has been active especially in antibiotic stewardship education [33], braving the first steps to promoting antibiotic stewardship programs in the country; steps that other nations with a similar pattern of antibiotic use should also follow.

## Limitation

In this retrospective study our data is limited by the accuracy and completeness of the medical records, therefore no inferences were made on immunization and vital signs because of this. We don’t have the exact timing of the laboratory draws, but all reported laboratory results were done during the sentinel ED visit. We also do not have information on the duration of antibiotic pretreatment, nor why it was given and therefore cannot determine its exact impact on cultures and laboratory results. In addition, the SBI rates of the pretreated group may be underreported as the antibiotics could have influenced the culture results. However, this reflects the reality of our clinical practice and decisions we have to make.

## Conclusions

In conclusion, this is the first study of its kind to describe febrile children already on antibiotics presenting to the ED compared to those not on antibiotics. It generated interesting preliminary data that opens doors to further investigations on predictors for testing febrile patients on antibiotics, on understanding how to interpret the test results and more importantly to understand predictors of SBI and SBI outcomes in this group.

## Supplementary information

**Additional file 1.**

## Data Availability

The datasets used and/or analysed during the current study are available from the corresponding author on reasonable request.

## Abbreviations

AAP: American Academy of Pediatrics
APUA: Alliance for the Prudent Use of Antibiotics
Cfu/ml: Colony forming unit/milliliter
CI: Confidence interval
ED: Emergency department
IM: Intramuscular
IV: Intravenous
OR: Odds ratio
SBI: Serious bacterial infection
SD: Standard deviation
SPA: Suprapubic aspiration
SPSS: Statistical Package for Social Sciences
URTI: Upper respiratory tract infection
UTI: Urinary tract infection
WBC: White blood cell
