# Supplementary material for: Comparing febrile children presenting on and off antibiotics to the emergency department: a retrospective cohort study
Source: BMC Pediatr. 2020 Mar 12;20:117. doi: 10.1186/s12887-020-2007-4 (PMC7069000; doi:10.1186/s12887-020-2007-4)
Supplement: Supplementary file 1 — Additional file 1. [file 12887_2020_2007_MOESM1_ESM.doc]

**Vital Signs Reference**

| **AGE** | **Est. Weight (Kg)** | **< HR** | **< RR** | **> SBP** |
| --- | --- | --- | --- | --- |
| **0-1m** | 3 | 160 | 60 | 60 |
| **1-3m** | 4 | 150 | 40 | 65 |
| **3-6m** | 6 | 150 | 40 | 65 |
| **6-9m** | 8 | 140 | 35 | 70 |
| **9-12m** | 10 | 140 | 35 | 70 |
| **1-3yr** | 12 | 130 | 30 | 74 |
| **3-5yr** | 16 | 120 | 30 | 76 |
| **5-7yr** | 20 | 110 | 25 | 80 |
| **7-9yr** | 24 | 110 | 20 | 86 |
| **≥ 10yr** | 30 | 100 | 18 | 100 |

**Limits of Normal**

**O2 Saturation >98 % at all ages**
